# A Multicenter International Retrospective Investigation Assessing the Prognostic Role of Inflammation-Based Scores (Neutrophil-to-Lymphocyte, Lymphocyte-to-Monocyte, and Platelet-to-Lymphocyte Ratios) in Patients with Intermediate-Stage Hepatocellular Carcinoma (HCC) Undergoing Chemoembolizations of the Liver

**DOI:** 10.3390/cancers16091618

**Published:** 2024-04-23

**Authors:** Roberto Minici, Massimo Venturini, Giuseppe Guzzardi, Federico Fontana, Andrea Coppola, Filippo Piacentino, Federico Torre, Marco Spinetta, Pietro Maglio, Pasquale Guerriero, Michele Ammendola, MGJR Research Team, Luca Brunese, Domenico Laganà

**Affiliations:** 1 Radiology Unit, University Hospital Dulbecco, 88100 Catanzaro, Italydomenico.lagana@unicz.it (D.L.); 2 Diagnostic and Interventional Radiology Unit, ASST Settelaghi, Insubria University, 21100 Varese, Italy; federico.fontana@uninsubria.it (F.F.); filippo.piacentino@asst-settelaghi.it (F.P.); 3 Imagerie Vasculaire et Interventionnelle, Centre Hospitalier Princesse Grace, 98000 Monaco, Monaco; giuguzzardi@gmail.com (G.G.); fede.torre85@gmail.com (F.T.); 4 Radiology Unit, Maggiore della Carità University Hospital, 28100 Novara, Italy; marcospinetta90@gmail.com; 5 Pain Management Unit, University Hospital Dulbecco, 88100 Catanzaro, Italy; pietromagliomd@gmail.com; 6 Department of Medicine and Health Sciences, University of Molise, 86100 Campobasso, Italy; pasqualeguerriero@gmail.com (P.G.); luca.brunese@unimol.it (L.B.); 7 Digestive Surgery Unit, University Hospital Dulbecco, 88100 Catanzaro, Italy; michele.ammendola@unicz.it

**Keywords:** tumor microenvironment, hepatocellular carcinoma (HCC), transcatheter arterial chemoembolization (TACE), drug-eluting microspheres (DEM), drug-eluting beads (DEB), lymphocyte-to-monocyte ratio (LMR), neutrophil-to-lymphocyte ratio (NLR), platelet-to-lymphocyte ratio (PLR), inflammation-based scores, prognostic marker

## Abstract

**Simple Summary:**

Inflammation-based scores reflect the intricate crosstalk between the tumor and the immune system, hosted in the tumor stromal microenvironment. In patients with hepatocellular carcinoma (HCC), these scores have shown promise in predicting recurrence, disease progression, and overall survival, as well as in forecasting the response to locoregional therapies. However, the specific predictive role of these inflammation-based scores in patients with intermediate-stage HCC undergoing TACE remains an area that requires further investigation. Early recognition of TACE refractoriness or failure holds the potential to guide tailored therapeutic interventions. Our research endeavors to fill a critical void in the existing literature by presenting, for the first time, data sourced from an international, multicenter study, involving Western institutions, thereby furnishing valuable insights applicable to this specific population. Our study demonstrates the prognostic value of inflammation-based scores, particularly Neutrophil-to-Lymphocyte Ratio (NLR) and Lymphocyte-to-Monocyte Ratio (LMR), in predicting the treatment response and short-term outcomes of patients with intermediate-stage HCC undergoing TACE.

**Abstract:**

Background: The utilization of inflammation-based scores, such as the Neutrophil-to-Lymphocyte Ratio (NLR), Lymphocyte-to-Monocyte Ratio (LMR), and Platelet-to-Lymphocyte Ratio (PLR), has garnered attention for their potential as prognostic indicators in various cancers. However, their predictive role in patients with intermediate-stage HCC undergoing transcatheter arterial chemoembolization (TACE) remains an area that requires further investigation, as early recognition of TACE refractoriness holds the potential to guide tailored therapeutic interventions. Methods: This multicenter international retrospective study analyzed data from patients with intermediate-stage HCC undergoing TACE between 2018 and 2024. Inflammation-based scores (NLR, LMR, PLR) were assessed preoperatively to predict treatment outcomes. Results: Two hundred and fourteen patients were enrolled. Preoperative LMR showed the largest area under the curve for the prediction of 6-months PFS, based on the ROC curve analysis. Both high LMR (≥2.24) and low NLR (<4.72) were associated with improved objective response rates and 6-month progression-free survival. Lymphocyte count emerged as a strong predictor of treatment response in both simple (*p* < 0.001) and multiple (*p* < 0.001) logistic regression analyses. Conclusions: This study highlights the prognostic value of inflammation-based scores, particularly LMR and NLR, in predicting the treatment response and short-term outcomes of patients with intermediate-stage HCC undergoing TACE. Future investigations should focus on validating these scores’ clinical applicability and assessing their impact on long-term patient survival and therapeutic decision-making.

## 1. Introduction

Hepatocellular Carcinoma (HCC) remains a significant global health burden, ranking as the third leading cause of cancer-related mortality worldwide [1]. Intermediate-stage HCC, classified under the Barcelona Clinic Liver Cancer (BCLC) staging system, often necessitates transcatheter arterial chemoembolization (TACE) as the primary therapeutic intervention [2,3]. However, within the intermediate stage, extremely diverse HCCs can be found in terms of morphological traits, recommended treatment, and prognosis [4]. Recent updates from the BCLC suggest instances where a left-to-right shift along the therapeutic algorithm is warranted, and, conversely, other scenarios where successful downstaging may lead to liver transplantation [4], resulting in post-transplant clinical outcomes akin to those of patients who underwent liver transplantation without prior progression beyond the early stage [5]. Therefore, it is evident that stage B HCCs can be extremely varied, exhibiting considerable heterogeneity in terms of tumor biology [6,7].

The utilization of inflammation-based scores, such as the Neutrophil-to-Lymphocyte Ratio (NLR), Lymphocyte-to-Monocyte Ratio (LMR), and Platelet-to-Lymphocyte Ratio (PLR), has garnered attention for their potential as prognostic indicators in various cancers [8]. These scores reflect the intricate crosstalk between the tumor and the immune system, hosted in the tumor stromal microenvironment [9]. In patients with HCC, these scores have shown promise in predicting recurrence, disease progression, and overall survival, as well as in forecasting the response to locoregional therapies (LRT) [10]. However, the specific predictive role of these inflammation-based scores in patients with intermediate-stage HCC undergoing TACE remains an area that requires further investigation. Early recognition of TACE refractoriness or failure through the utilization of inflammation-based scores holds the potential to guide tailored therapeutic interventions, ultimately leading to improved outcomes for patients with intermediate-stage HCC [10]. 

The sole investigation that comparatively assessed NLR, LMR, and PLR in intermediate-stage HCC patients was carried out by Liu and colleagues [11]. Nonetheless, this study was conducted at a single center and predominantly enrolled individuals of Asian descent. The recognized biological and prognostic diversity across various medical conditions, including HCC, is well-documented, with substantial implications varying among different ethnic backgrounds [12]. Our study, a multicenter international retrospective investigation, aims to assess the prognostic role of inflammation-based scores (namely, NLR, LMR, and PLR) in patients with intermediate-stage HCC undergoing chemoembolizations of the liver. Our research endeavors to fill a critical void in the existing literature by presenting, for the first time, data sourced from an international, multicenter study, involving Western institutions, thereby furnishing valuable insights applicable to this specific population.

## 2. Materials and Methods

### 2.1. Study Design

This research constitutes an international, multi-center (Mater-Domini center of the Dulbecco University Hospital, Catanzaro, Italy; Circolo Hospital, Varese, Italy; Maggiore della Carità University Hospital, Novara, Italy; Centre Hospitalier Princesse Grace, Monaco, Principality of Monaco) retrospective analysis of prospectively gathered data from patients diagnosed with intermediate-stage HCC. The study, conducted between January 2018 and February 2024, included patients who underwent either drug-eluting microspheres (DEM)-TACE or conventional TACE (cTACE) as their primary treatment. The criteria for inclusion encompassed several factors: (I) DEM-TACE or cTACE for BCLC stage B HCC [4]; (II) HCC diagnosis according to the European Association for the Study of the Liver criteria [3]; (III) a Child–Pugh score of up to 9; (IV) absence of prior HCC treatment; (V) Eastern Cooperative Oncology Group performance status grade 0 [13]; (VI) evaluation by a multidisciplinary team (hepatologist, liver surgeon, and interventional radiologist). Exclusion criteria were also defined, including missed imaging follow-up, abnormal serum creatinine (i.e., >2 mg/dL) or bilirubin (i.e., >3 mg/dL) levels, platelet count <50,000/μL, international normalized ratio (INR) >1.5, contraindications for doxorubicin administration, previous TACE, high-flow arterioportal or arteriovenous shunts, clinical and/or laboratory signs of infection or inflammation, and a Child–Pugh score > B9. Ethical committee approval was not required since the study was retrospective. Ethical standards were maintained in line with the Declaration of Helsinki. All patients signed an informed consent form before undergoing TACE.

### 2.2. Treatment

TACE followed the technical steps outlined in the previous literature [14,15], with its essential features summarized as follows. Arterial access via either the radial or femoral route was established according to the operator’s preference, possessing over 5 years of experience. After selectively catheterizing the common hepatic artery with a 4 or 5 French diagnostic catheter, digital subtraction angiography (DSA) was conducted. Subsequent DSA imaging was performed from the proper hepatic artery after its selective catheterization with a 2.7 French microcatheter (Progreat, Terumo, Japan). Identification of tumor feeders and their superselective catheterization were performed. DEM-TACE utilized PEG-based microspheres sized 200 ± 50 μm, loaded with 75 mg of doxorubicin and mixed with iodinated contrast. Administration of the drug ceased once stasis was maintained for at least 10 cardiac beats [16]. cTACE was performed adhering to the technique previously outlined in the Standard of Practice by the Cardiovascular and Interventional Radiological Society of Europe (CIRSE) [15]. The choice between DEM-TACE or cTACE was made based on the operator’s discretion. Each patient underwent clinical, laboratory, and imaging follow-up at 1 month and 3 months post-procedure, and subsequently every 3 months. Contrast-enhanced CT or gadolinium-enhanced MRI was utilized for follow-up imaging purposes.

### 2.3. Outcomes and Definitions

The primary outcome is the ability of preoperative NLR, LMR, and PLR to predict PFS at 6 months. The ability of preoperative NLR, LMR, and PLR to predict complete response (CR), objective response (OR), sustained response duration (SRD) exceeding 6 months, successful downstaging at 6 months, and overall survival (OS) at 6 months defined the secondary outcomes.

LMR was calculated as the ratio of the absolute count of lymphocytes (number of lymphocytes/µL) to the absolute count of monocytes (number of monocytes/µL). NLR was calculated as the ratio of the absolute count of neutrophils (number of neutrophils/µL) to the absolute count of lymphocytes (number of lymphocytes/µL). PLR was calculated as the ratio of the absolute count of platelets (number of platelets/µL) to the absolute count of lymphocytes (number of lymphocytes/µL). The preoperative laboratory assessment was performed within 48 h prior to TACE. CR and OR were assessed during the 1-month imaging follow-up. SRD, successful downstaging, PFS, and OS were evaluated 6 months after the first TACE procedure. Technical success was determined by the complete delivery of the planned doxorubicin dose and achieving a cessation of blood flow for at least 10 cardiac beats, in accordance with the standard of practice set by the CIRSE [15]. Treatment response was evaluated based on modified Response Evaluation Criteria In Solid Tumors (mRECIST) guidelines [17]. CR was defined as the absence of arterial enhancement within all target lesions. Partial Response (PR) was characterized by at least a 30% reduction in the sum of the diameters of viable (contrast-enhancing) target lesions. Progressive Disease (PD) was identified by a minimum 20% increase in the sum of the diameters of viable (enhancing) target lesions, whereas Stable Disease (SD) included cases not meeting the criteria for PR or PD. Patients exhibiting new lesions, vascular invasion, and/or metastases were classified as having PD. Disease control was calculated as the sum of CR, PR, and SD [18,19]. Objective response comprised patients who achieved either CR or PR. Sustained response duration was defined as the duration between the date of achieving CR, PR, or SD and the date of progression.

### 2.4. Statistical Analysis

Data were documented and arranged within a Microsoft Excel spreadsheet (Microsoft Inc, Redmond, WA). Subsequent statistical analyses were carried out utilizing SPSS software (SPSS, version 26 for Windows; SPSS Inc., Chicago, IL, USA). Our investigation centered on the per-protocol population, consisting of all randomly assigned patients who underwent a chemoembolization procedure and fulfilled imaging follow-up requirements. The normality assumption of the data was assessed using the Kolmogorov–Smirnov and Shapiro–Wilk tests [20]. Categorical data were presented as frequency (percentage value) [21], while continuous, normally distributed data were expressed as mean ± standard deviation [22]. Continuous data not conforming to a normal distribution were represented as the median (interquartile range: 25th and 75th percentiles—IQR) [23]. Statistical variances for continuous, normally distributed data were assessed using the unpaired Student *t*-test [24], whereas the Chi-squared or Fisher’s exact test [25] was utilized for categorical data. The Mann–Whitney test was employed for continuous data not meeting normal distribution assumptions. Cut-off values for NLR, LMR, and PLR were established using the receiver operating characteristic (ROC) curve for 6-month PFS [26]. The optimal cut-off points, maximizing sensitivity and specificity, were determined by the best area under the curve (AUC). Simple and multiple logistic regression analyses were conducted to assess potential factors predicting the occurrence of OR and 6-month PFS [27]. Variables demonstrating a significance level of *p* < 0.05 in univariable analyses were incorporated into the multivariable logistic regression analyses [28].

## 3. Results

**Figure 1:**
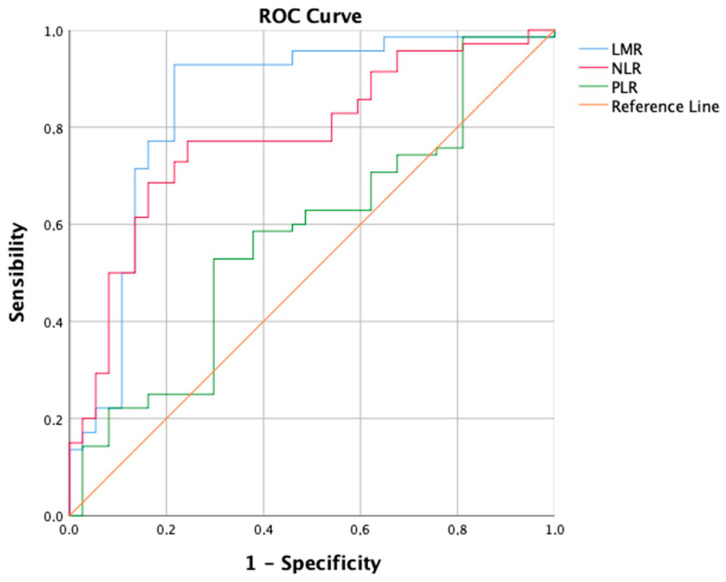
Receiver operating characteristic (ROC) curve showing the predictive values of Lymphocyte-to-monocyte ratio (LMR), Neutrophil-to-lymphocyte ratio (NLR), and Platelet-to-lymphocyte ratio (PLR), for 6-month Progression-free Survival (PFS).

**Figure 2:**
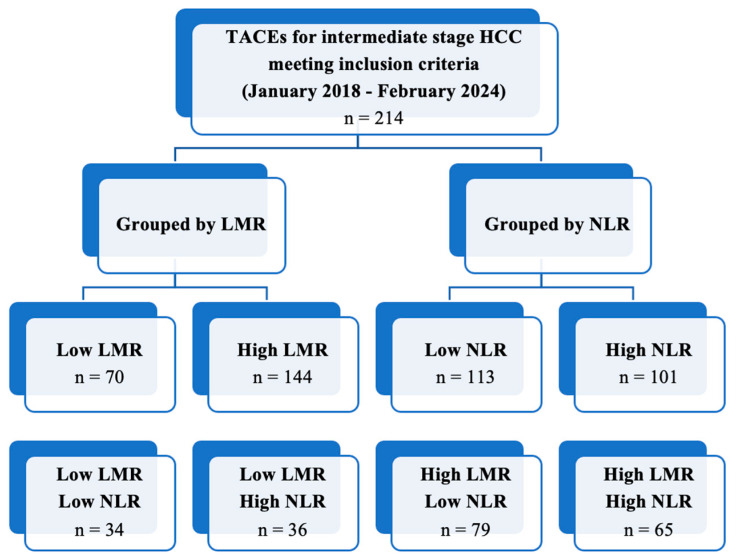
Flowchart depicting the study population grouped by LMR and NLR.

TACE was performed as the first-line treatment in 214 consecutive patients with intermediate-stage HCC. A ROC curve (Figure 1) for the prediction of 6-months PFS was plotted to evaluate the performance of three binary classifier models (LMR, NLR, and PLR) at varying threshold values. Based on the ROC curve analysis, a cut-off value of 2.24 (sensibility, 0.931; specificity, 0.784) was chosen to divide the population into a low LMR group (Group 1—LMR < 2.24, *n* = 70, 32.7%) and a high LMR group (Group 2—LMR ≥ 2.24, *n* = 144, 67.3%). Similarly, a cut-off value of 4.72 (sensibility, 0.771; specificity, 0.757) was chosen to divide the population into a high NLR group (Group 1—NLR ≥ 4.72, *n* = 113, 52.8%) and a low NLR group (Group 2—NLR < 4.72, *n* = 101, 47.2%). Furthermore, a cut-off value of 119.67 (sensibility, 0.529; specificity, 0.703) was chosen to divide the population into a high PLR group (Group 1—PLR ≥ 119.7, *n* = 118, 55.1%) and a low PLR group (Group 2—PLR < 119.7, *n* = 96, 44.9%). The overall performance of the ROC curve was defined by an area under the curve (AUC) of 0.848 (CI: 0.785–0.911; SE: 0.032) for LMR, 0.779 (CI: 0.715–0.843; SE: 0.033) for NLR, and 0.577 (CI: 0.496–0.658; SE: 0.041) for PLR. Furthermore, a flowchart depicting the study population grouped by LMR and NLR has been drawn (Figure 2).

Table 1 and Table 2 present the baseline demographic and clinical data of 214 patients, categorized by LMR and NLR, respectively. Group 1 comprises patients with low LMR or high NLR, while Group 2 consists of those with high LMR or low NLR, respectively. Differences in the distribution of baseline data between the two groups were tested for each inflammation-based score used (namely, LMR or NLR). Noteworthy distinctions emerged, with both high LMR and low NLR groups exhibiting a markedly higher lymphocyte count, higher LMR, and lower NLR. The high LMR group, but not low NLR group, showed lower alpha-fetoprotein, higher albumin, lower neutrophil count, lower monocyte count, and lower PLR.

**Figure 3:**
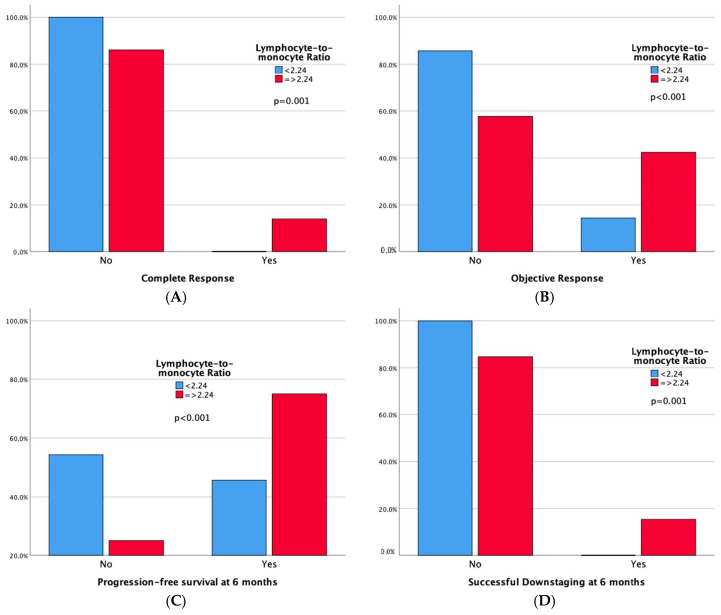
Bar plots representing Complete Response (**A**), Objective Response (**B**), Progression-free Survival at 6 months (**C**), and Sustained Response Duration ≥ 6 months (**D**), according to LMR Groups. The *p*-values pertain to the comparison of outcome frequencies between the two subgroups (Low LMR vs. High LMR).

**Figure 4:**
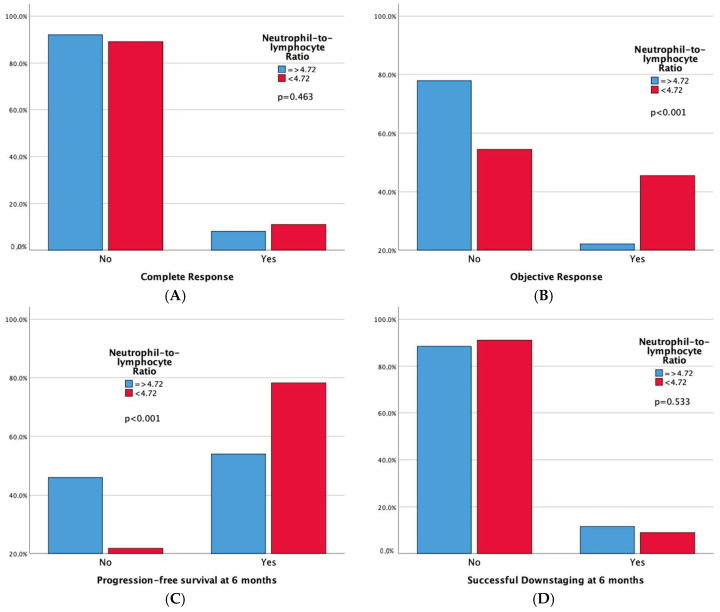
Bar plots representing Complete Response (**A**), Objective Response (**B**), Progression-free Survival at 6 months (**C**), and Sustained Response Duration ≥ 6 months (**D**), according to NLR Groups. The *p*-values pertain to the comparison of outcome frequencies between the two subgroups (Low NLR vs. High NLR).

Technical success rates were high (100%) in both groups. Higher complete response (CR) and 6-month successful downstaging rates were noted in the high LMR group, but not in the low NLR group. Significant differences emerged in tumor response, with both high LMR and low NLR groups demonstrating superior 6-month progression-free survival rates and overall objective response. Moreover, adverse events and a sustained response duration ≥ 6 months did not exhibit significant differences between both the LMR and NLR groups. Outcomes data are summarized in Table 3 and Table 4. Furthermore, bar plots are provided to depict differences in Complete Response, Objective Response, Progression-free Survival at 6 months, and Sustained Response Duration ≥ 6 months, between the low vs. high LMR groups (Figure 3) and between the high vs. low NLR groups (Figure 4).

Simple logistic regression analyses showed that age, Hepatitis C virus, α-Fetoprotein, lymphocyte count, monocyte count, NLR, NLR Groups (<4.72), LMR and LMR groups (≥2.24) were significant single predictors of Objective Response occurrence. White blood cell count, LMR, and NLR were excluded from the multiple logistic regression analysis due to their possible interference effect with lymphocyte, neutrophil, and monocyte counts, thus maintaining the independence of the tested variables. Multiple logistic regression analyses showed that age, Hepatitis C virus, α-Fetoprotein, and lymphocyte count were significant multiple predictors of Objective Response occurrence. Interestingly, it is noted that an increase of one hundred units in the lymphocyte count is associated with an average increase of 0.4 in the log-odds of Progression-free survival at 6 months. The percentage accuracy in the classification of the multiple binomial logistic regression model is 83.2%, meaning that 83.2% of Objective Response instances can be correctly classified with the independent variables added. Details are reported in Table 5.

Simple logistic regression analyses showed that α-Fetoprotein, white blood cell count, neutrophil count, lymphocyte count, monocyte count, NLR, NLR Groups (<4.72), LMR, and LMR groups (≥2.24), were significant single predictors of Progression-free Survival at 6 months. White blood cell count, LMR, and NLR were excluded from the multiple logistic regression analysis due to their possible interference effect with lymphocyte, neutrophil, and monocyte counts, thus maintaining the independence of the tested variables. Interestingly, lymphocyte and monocyte counts were found to be significant predictors of Progression-free Survival at 6 months. It is noted that a unit increase in the lymphocyte count is associated with an average increase of 0.003 in the log-odds of Progression-free survival at 6 months. The percentage accuracy in classification of the multiple binomial logistic regression model is 84.1%, meaning that 84.1% of 6-month Progression-free Survival instances can be correctly classified with the independent variables added. Details are given in Table 6.

## 4. Discussion

Our study’s key findings can be summarized as follows:

- In patients with intermediate-stage HCC undergoing TACE, LMR demonstrates a good predictive value for short-term outcomes like 6-month PFS, while NLR shows a moderate accuracy according to ROC analysis. Conversely, PLR’s predictive performance is only marginally better than random chance.
- One hundred-and-forty-four patients have a high LMR (≥2.24), also exhibiting better objective response (42.4% vs. 14.3%, *p* < 0.001) and 6-month PFS (75% vs. 45.7%, *p* < 0.001) rates compared to patients with low LMR. One hundred-and-one patients have a low NLR (<4.72), also showing better objective response (45.5% vs. 22.1%, *p* < 0.001) and 6-month PFS (78.2% vs. 54%, *p* < 0.001) rates compared to patients with high NLR. Notably, high LMR alone is also linked to higher Complete Response (13.9% vs. 0%, *p* = 0.001) and 6-month successful downstaging (15.3% vs. 0%, *p* = 0.001) rates.
- Among the leukocyte components comprising LMR and NLR, only lymphocyte count remains a robust predictor of Objective Response in both simple and multiple logistic regression analyses. Furthermore, in the multiple logistic regression model, both lymphocyte and monocyte counts significantly predict 6-month PFS.

In response to the diverse patient population in intermediate-stage HCC, the need for further subclassification has been accounted by the 2022 BCLC update to customize optimal therapy for each patient [4]. Interestingly, patients meeting the up-to-seven criteria with well-preserved liver function tend to derive the most benefit from TACE, with the potential for downstaging in some cases. Conversely, patients with extensive tumor burdens beyond the up-to-seven criteria typically undergo multiple TACE cycles until they experience TACE failure or refractoriness, which negatively impacts liver function following each procedure [7]. An analysis of the RESORCE trial showed that sequential sorafenib–regorafenib treatment resulted in favorable overall survival (26 vs. 19.2 months) in the regorafenib arm compared to the placebo arm, starting from sorafenib initiation to death. This prolonged survival observed with sorafenib–regorafenib sequential therapy in predominantly BCLC C patients (86% of the RESORCE trial) suggests that applying this sequential regimen in BCLC B TACE-refractory patients could potentially achieve a survival exceeding 26 months, similar to conventional TACE therapy outcomes [29]. Repeated TACE in TACE-refractory patients diminishes liver function without improving survival and may affect their eligibility for subsequent systemic therapy. Therefore, promptly transitioning from repeated TACE to systemic therapy and/or radioembolization in TACE-refractory patients may enhance their overall survival and facilitate liver transplantation post-successful downstaging [30]. Understanding tumor biology is crucial for delivering timely personalized treatments to intermediate-stage HCC patients. Various studies have indicated that the response to locoregional treatments like TACE can serve as an indicator of tumor biology [31,32]. However, TACE refractoriness or failure often emerges as a late-stage marker due to routine quarterly imaging after the first 30-day follow-up [15], thus resulting in critical time loss. Inflammation-based scoring systems, such as the NLR, the LMR, and the PLR, act as markers of the complex interplay between the cancer biology and immune system [8,9]. These scoring systems are reliable and easily accessible preoperative markers, that could predict response to therapy and clinical outcomes in HCC patients [33,34,35]. To the best of our knowledge, our report represents the first multinational, multicenter study in Western countries to demonstrate that inflammation-based scores effectively predict therapy response and short-term clinical outcomes in patients with intermediate-stage HCC undergoing TACE. Better predictive performances of LMR compared to NLR and PLR were noted. Early prediction of TACE response could allow for tailoring patient treatment pathways to tumor biology, potentially leading to survival benefits. However, this remains speculative and beyond the scope of our study, warranting further investigation in the future. Our report findings are consistent with those of other investigations assessing inflammation-based scores in HCC patients undergoing surgical or locoregional treatments. Lin et al. found that the LMR was an independent predictor of OS and recurrence-free survival (RFS) in HBV-associated HCC patients after hepatectomy [36]. Wang et al. explored the prognostic significance of the monocyte-to-lymphocyte ratio in HCC patients undergoing combined treatment with TACE and ablative therapy, demonstrating its ability to predict early relapse and survival [37]. Additionally, studies have shown that elevated LMR is associated with extended OS in patients treated with radiofrequency ablation (RFA) plus TACE [38], and that a combination of low NLR and high LMR predicts better OS after TACE [39]. In a recent meta-analysis, Li et al. concluded that elevated preoperative NLR and PLR are associated with poor prognosis in HCC patients treated with TACE [40]. High baseline NLR effectively predicts OS and time-to-progression (TTP) in patients specifically treated with cTACE [41]. Schobert et al. demonstrated an effective predictive role of PLR and NLR for patients treated with drug-eluting beads (DEB)-TACE [42]. It is worth noting that the literature on the predictive capabilities of PLR is quite heterogeneous. Cho et al. highlighted that PLR is not a reliable predictor of progression, as evidenced by a multiple logistic regression analysis of a 605-patient cohort undergoing TACE [41]. According to Itoh et al., LMR, but not NLR and PLR, emerges as a strong independent predictor of OS and RFS in patients undergoing hepatic resection for HCC [43]. Conversely, our study diverges notably from Liu et al., who identified PLR as the foremost inflammation-based score for predicting PFS in intermediate-stage HCC patients undergoing TACE [11]. One reason for this disparity lies in the different ethnicities prevalent in the populations studied. Genetic factors are known to wield significant influence over tumor biology and therapy response [12]. Additionally, variations in ALBI grade prevalence and mean alpha-fetoprotein levels between our study and Liu et al.’s may reflect a different severity of cirrhosis and its influence on platelet levels, thus introducing a confounding factor. Lastly, while our study employed logistic regression for evaluating predictors, Liu et al. opted for time-dependent Cox regression. Our study brings several advantages compared to prior research. Firstly, it is an international, multicenter endeavor, making its findings relevant to Caucasian patients, a departure from the majority of studies confined to Eastern populations. Secondly, we directly assess and compare the three key inflammation-based scores previously explored in HCC literature (i.e., NLR, LMR, and PLR). Thirdly, we establish cut-off values through ROC curve analysis, differently from other studies relying on median or mean values [34]. Lastly, our focus solely on intermediate-stage HCC patients undergoing TACE sets us apart from studies examining combined treatments (e.g., RFA plus TACE), such as that of Shen et al. [38], thus avoiding potential confounding factors.

The comparison of ROC curves revealed that the LMR model demonstrates superior diagnostic performance in predicting PFS at 6 months as a binary classifier [44]. Consistent with findings by Muller et al. [45], the LMR’s predictive role, with an AUC of 0.848, is considered good, while NLR shows moderate discriminatory ability (AUC = 0.779) and PLR performs only slightly better (AUC = 0.577) than random chance. In a similar vein, Liu et al. employed the ROC curve to assess 3-month PFS, identifying a cut-off of 2.20 for LMR with an AUC of 0.751, and 3.94 for NLR with an AUC of 0.845 [39]. However, Shen et al. did not specify the clinical outcome for which the ROC curve was constructed. They opted for cut-off values of 2.13 for LMR (AUC = 0.639) and 95.65 for PLR (AUC = 0.731). Surprisingly, the NLR cut-off was not selected, yielding an AUC of only 0.617 [38]. Interestingly, the strong biological rationale for the limited discriminatory capability of PLR lies in the prevalence of chronic liver disease (CLD) and thrombocytopenia in hepatocellular carcinoma patients. Platelet levels are influenced by various factors beyond the tumor microenvironment and immune system interplay. Historically, thrombocytopenia was traditionally associated with hypersplenism, a condition characterized by the accumulation of platelets in an enlarged spleen due to portal hypertension-induced congestive splenomegaly [46]. However, recent years have witnessed significant progress in the understanding of thrombopoiesis, leading to a more nuanced comprehension of thrombocytopenia in cirrhosis. Various factors contribute to thrombocytopenia, encompassing reduced production, splenic sequestration, and enhanced destruction. Diminished thrombopoietin levels in chronic liver disease (CLD), coupled with direct bone marrow suppression, lead to decreased platelet production rates [47]. Thrombopoietin plays a crucial role in platelet production and maturation, and its functionality is compromised in CLD [48]. Viruses, alcohol, iron overload, and medications can induce bone marrow suppression [49]. Splenic sequestration is a consequence of hypersplenism [47]. The accelerated platelet destruction in cirrhosis is mediated through multiple pathways: heightened shear stress, increased fibrinolysis, bacterial translocation, and infections contribute to elevated platelet aggregation rates, while autoimmune disorders and elevated levels of antiplatelet antibodies lead to immunological platelet destruction [50,51,52,53]. Therefore, based on a deep understanding of the intricate pathophysiological mechanisms governing thrombocytopenia in liver disease patients, our study argues that PLR is an inadequate predictor of short-term outcomes like 6-month PFS in stage B HCC patients undergoing TACE.

The method for selecting optimal cut-off values of inflammation-based scores varies widely among studies, introducing potential biases and sources of confounding [10]. In some instances, the mean or median of the study population has been employed [54,55], but this does not appear to be an optimal choice as it is entirely detached from the predictive function being tested. Most commonly, the ROC curve has been utilized, which evaluates a biomarker’s capacity to classify disease status [44]. The selection of the optimal cut-off aims to simultaneously maximize sensitivity and specificity, but the shape of the ROC curve does not always facilitate the straightforward identification of such a value [56]. In certain cases, as demonstrated by Wang et al., the Youden index can be employed, defining the segment with the maximum distance between the chance line and the ROC curve [37,56]. In our study, we utilized the ROC curve, the shape of which easily allowed us to pinpoint the optimal cut-off value of LMR at 2.24 for classifying 6-month PFS. In a report on 210 HBV-associated HCC patients undergoing liver resection, the ROC curve analyses for OS indicated the optimal LMR cutoff value. The prognostic impact of inflammation-based scores was evaluated through univariate and multivariate analyses, using the Cox proportional hazards model [36]. Previous studies on HCC patients undergoing chemoembolization had identified similar cut-off values. Specifically, Shen et al. identified a cut-off of 2.13 in patients undergoing cTACE plus RFA [38], while Liu et al. opted for a cut-off of 2.2 in a cohort of 180 patients with large HCC undergoing cTACE [39]. Ultimately, we recommend avoiding methods such as median or mean and to use a method like the ROC curve to select the optimal cut-off value of inflammation-based scores for prediction purposes.

Interestingly, to delve into the reasons behind the superior predictive abilities of LMR compared to NLR, it is beneficial to individually examine the specific components of the leukocyte formula that make up these inflammation-based scores. High LMR and low NLR are both significantly associated with improved Objective Response rates. However, only the absolute lymphocyte count (ALC) among the leukocyte formula components remains a strong predictor of Objective Response in multiple logistic regression. Additionally, the low NLR group has a notably higher ALC than the high NLR group, while the absolute neutrophil count remains consistent. This emphasizes the crucial role of the ALC in influencing NLR and predicting outcomes, as supported by ROC curve analysis and multiple logistic regression. Similarly, in their study involving 210 patients with HCC who underwent curative resection, Lin et al. demonstrated that the ALC count served as a significant predictor of overall survival [36]. In contrast, in our investigation, the absolute neutrophil count does not play a significant predictive role in any of the multiple logistic regression models incorporating leukocyte formula parameters. The absolute monocyte count falls in between, showing significant differences between high and low LMR groups and proving to be a negative predictor of 6-month PFS but not Objective Response rate in a multiple logistic regression model. Therefore, our study underscores the pivotal role of the ALC among leukocyte formula components in predicting and prognosticating based on the tested inflammation-based scores. The biological plausibility of these findings should be considered in the context of the roles of lymphocytes, neutrophils, macrophages, and platelets in the tumor microenvironment (TME).

The tumor microenvironment (TME) comprises blood and lymph vessels, cytokines, extracellular vesicles, extracellular matrix, and non-cancerous cells like T cells, adipocytes, neutrophils, macrophages, and stromal cells [57]. Lymphocytes play a crucial role in immune surveillance and response within the TME, influencing the body’s immune reaction against cancer [58]. The presence of tumor-infiltrating lymphocytes (TILs) is linked to better outcomes across various cancers including HCC, while low lymphocyte levels and failure to penetrate the tumor are associated with poorer survival rates [59,60,61]. Tumor-specific antigens can be identified by T lymphocytes infiltrating the tumor, stimulating an anti-tumor immune response [62]. Cancer cells hinder the proliferation of cytotoxic T lymphocytes (CTLs) within the tumor through the production of immunosuppressive cytokines like interleukin (IL)-10, vascular endothelial growth factor (VEGF), and TGF-β, and by consuming IL-2, a critical cytokine for CTL function [63]. A report by Unitt et al. revealed that decreased lymphocyte infiltration and a low CD4+/CD8+ T cell ratio were independent predictors of HCC recurrence post-liver transplantation [64]. Other studies showed that low levels of intratumoral cytotoxic CD8+ T cells and high levels of intratumoral regulatory T cells were linked to worse prognoses in HCC patients post-resection [65,66]. Hence, the ALC could serve as a simple surrogate marker of immune response [36]. Neutrophils have garnered attention for their role in promoting cancer initiation, progression, and metastases. They contribute to carcinogenesis by heightening inflammation pathways, cause DNA damage through genotoxic substances, and foster neoangiogenesis and immunosuppression [67,68]. Activated neutrophils release neutrophil extracellular traps that exacerbate inflammation in CLD, promoting the onset of HCC, and also facilitate naive CD4+ T cells metabolic reprogramming, correlating positively with regulatory T cell numbers in cancer [69,70]. Cytokines produced by cancer cells in the TME, like transforming growth factor-β (TGFβ), can alter the cancer microenvironment by reshaping neutrophils into either a cancer-promoting (N2) or antitumor (N1) phenotype [71]. Circulating monocytes are attracted to the tumor stroma and transform into tumor-associated macrophages (TAM) in response to tumor-released chemokines [72]. TAMs secrete growth factors and cytokines to influence the tumor microenvironment, thereby facilitating tumor angiogenesis, progression, and metastasis [73,74]. The adverse impact of monocytes on HCC prognosis has been linked to poor outcomes, as evidenced by studies conducted by Sasaki et al. [75], Shen et al. [76], and Lin et al. [36], who found that monocytosis was correlated with reduced OVS in HCC patients post-resection. Our results are consistent with these previous reports. The assessment of TAMs can be performed using peripheral blood monocytes, serving as a biological indicator [36,77].

The potential limitations of this study necessitate cautious consideration in interpreting its findings. Firstly, the study did not assess statistical power upfront due to its retrospective nature, and the 214 patients enrolled can be considered a relatively small sample size. Therefore, the lack of statistical significance in some findings could be attributed to a type II error (i.e., failing to reject a false null hypothesis) [78]. While acknowledging that the absence of evidence for certain findings does not necessarily imply the proof of absence of statistical significance, future directions may involve conducting prospective observational studies with larger sample sizes and pre-calculated statistical power. Secondly, selection bias might be introduced due to the retrospective nature of the study. Thirdly, an in-depth examination of the tumor microenvironment was not formally conducted. Fourthly, the study’s reliance on a limited sample size and its exclusive focus on short-term outcomes may restrict the broader applicability of the results. Furthermore, we did not compare the subgroup with low LMR and high NLR to the subgroup with high LMR and low NLR because of the small sample sizes in these groups. Therefore, future plans involve conducting a subgroup analysis where LMR and NLR are assessed together, rather than separately. Lastly, the potential clinical utility of inflammation-based scores to alter treatment strategies and positively influence patient survival remains speculative and necessitates evaluation through dedicated studies.

## 5. Conclusions

In conclusion, our study underscores the prognostic value of preoperative inflammation-based scores, particularly LMR and NLR, in intermediate-stage HCC patients receiving TACE. According to the ROC curve analysis, the predictive accuracy of LMR surpasses that of NLR, while the performance of PLR is notably inadequate. High LMR and low NLR correlate with improved objective response rate and 6-month progression-free survival. Among the leukocyte components comprising LMR and NLR, only lymphocyte count remains a robust predictor of objective response in both simple and multiple logistic regression analyses, thus demonstrating its pivotal role in determining the predictive capacity of inflammation-based scores. Future investigations should focus on validating these scores’ clinical applicability and assessing their impact on long-term patient survival and therapeutic decision-making.

## Figures and Tables

**Table 1:** Baseline demographic and clinical data, grouped by LMR.

Variables	All Patients (*n* = 214)			
		Group 1 Low LMR (*n* = 70)	Group 2 High LMR (*n* = 144)	*p* Value
Age (years)	57.2 (±13.7)	54.4 (±14.1)	58.6 (±13.4)	0.025
Sex (Female)	75 (35%)	30 (42.9%)	45 (31.3%)	0.095
Body Mass Index	26.6 (±5)	26.2 (±5)	26.8 (±5)	0.411
Smoking history	146 (68.2%)	47 (67.1%)	99 (68.7%)	0.813
Hepatitis B virus	28 (13.1%)	8 (11.4%)	20 (13.9%)	0.617
Hepatitis C virus	91 (42.5%)	14 (20%)	77 (53.5%)	<0.001
Non-alcoholic fatty liver disease	27 (12.6%)	11 (15.7%)	16 (11.1%)	0.341
Alcoholic liver disease	73 (34.1%)	33 (47.1%)	40 (27.8%)	0.005
α-Fetoprotein (ng/mL)	237.8 (±226.2)	311.9 (±284.6)	201.7 (±182)	0.026
Carbohydrate antigen 19-9 (U/mL)	11.5 (±13.6)	12.3 (±13.6)	11.2 (±13.6)	0.836
γ-Glutamyltransferase (U/L)	89.1 (±56.7)	91.8 (±70.9)	87.8 (±48.4)	0.860
Alkaline phosphatase (U/L)	52.6 (±21.9)	54.3 (±20.8)	51.8 (±22.4)	0.161
Aspartate transaminase (U/L)	63.6 (±28.2)	57.7 (±29.1)	66.5 (±27.4)	0.166
Alanine transaminase (U/L)	68.3 (±24.8)	70.3 (±25.6)	67.3 (±24.5)	0.227
Albumin (g/L)	30.1 (±2.7)	29.5 (±2.4)	30.4 (±2.8)	0.027
Total bilirubin (mg/dL)	1.12 (±0.4)	1.05 (±0.4)	1.15 (±0.4)	0.048
Direct bilirubin (mg/dL)	0.35 (±0.4)	0.34 (±0.4)	0.36 (±0.4)	0.742
Indirect bilirubin (mg/dL)	0.77 (±0.4)	0.71 (±0.4)	0.79 (±0.4)	0.189
Prothrombin time (seconds prolonged)	6.3 (±1.2)	6.1 (±1.2)	6.4 (±1.3)	0.136
Ascites	0 (0%)	0 (0%)	0 (0%)	NA
Child–Pugh score, A6/B7/B8/B9	10 (4.7%)/72 (33.6%)/128 (59.8%)/4 (1.9%)	0 (0%)/26 (37.1%)/42 (60%)/2 (2.9%)	10 (6.9%)/46 (31.9%)/86 (59.7%)/2 (1.4%)	0.123
ALBI grade, 1/2/3	20 (9.3%)/186 (86.9%)/8 (3.8%)	6 (8.6%)/61 (87.1%)/3 (4.3%)	14 (9.7%)/125 (86.8%)/5 (3.5%)	0.927
Cirrhosis	213 (99.5%)	69 (98.6%)	114 (100%)	0.151
Total cholesterol (mg/dL)	215.9 (±53.9)	214.5 (±53.3)	216.7 (±54.1)	0.779
Triglycerides (mg/dL)	105.5 (±22)	105.2 (±22)	105.7 (±22)	0.876
Platelet count (No. ×103/μL)	134.2 (±49.5)	128.5 (±42.8)	137 (±52.3)	0.502
Hemoglobin (g/dL)	11.5 (±1.43)	11.8 (±1.47)	11.4 (±1.40)	0.077
White blood cell count (per μL)	4649.4 (±823.5)	4771 (±717.4)	4590.3 (±866.5)	0.043
Neutrophil count (per μL)	3342.4 (±774.6)	3604.9 (±742.4)	3214.7 (±760)	<0.001
Lymphocyte count (per μL)	896.9 (±321.6)	710.8 (±285.5)	987.3 (±299.2)	<0.001
Monocyte count (per μL)	247.2 (±81.2)	277.1 (±78.6)	231.7 (±78.7)	<0.001
Lymphocyte-to-monocyte Ratio (LMR)	4.20 (±2.27)	2.82 (±1.57)	4.87 (±2.26)	<0.001
Neutrophil-to-lymphocyte Ratio (NLR)	4.43 (±2.61)	6.14 (±3.39)	3.60 (±1.57)	<0.001
Platelet-to-lymphocyte Ratio (PLR)	169.46 (±88.01)	210.73 (±106.11)	149.4 (±69.75)	<0.001
Maximum tumor size (cm)	4.49 (±1.13)	4.42 (±1.09)	4.53 (±1.16)	0.557
Bilobar disease	85 (39.7%)	29 (41.4%)	56 (38.9%)	0.722
Capsule	112 (52.3%)	40 (57.1%)	72 (50%)	0.326

**Table 2:** Baseline demographic and clinical data, grouped by NLR.

Variables	All Patients (*n* = 214)			
		Group 1 High NLR (*n* = 113)	Group 2 Low NLR (*n* = 101)	*p* Value
Age (years)	57.2 (±13.7)	57.5 (±15)	56.8 (±12.2)	0.455
Sex (Female)	75 (35%)	35 (31%)	40 (39.6%)	0.186
Body Mass Index	26.6 (±5)	26.6 (±5)	26.7 (±5)	0.884
Smoking history	146 (68.2%)	74 (65.5%)	72 (71.3%)	0.363
Hepatitis B virus	28 (13.1%)	10 (8.8%)	18 (17.8%)	0.052
Hepatitis C virus	91 (42.5%)	32 (31.7%)	59 (52.2%)	0.002
Non-alcoholic fatty liver disease	27 (12.6%)	11 (9.7%)	16 (15.8%)	0.179
Alcoholic liver disease	73 (34.1%)	38 (33.6%)	35 (34.7%)	0.875
α-Fetoprotein (ng/mL)	237.8 (±226.2)	258 (±248.7)	215.1 (±196.8)	0.118
Carbohydrate antigen 19-9 (U/mL)	11.5 (±13.6)	12.9 (±14.2)	10 (±12.8)	0.550
γ-Glutamyltransferase (U/L)	89.1 (±56.7)	91.3 (±52.9)	86.6 (±60.7)	0.379
Alkaline phosphatase (U/L)	52.6 (±21.9)	52.5 (±20.7)	52.6 (±23.3)	0.495
Aspartate transaminase (U/L)	63.6 (±28.2)	64 (±30.4)	63.2 (±25.7)	0.424
Alanine transaminase (U/L)	68.3 (±24.8)	71.1 (±26.2)	65.2 (±22.9)	0.058
Albumin (g/L)	30.1 (±2.7)	30.3 (±2.8)	29.9 (±2.6)	0.382
Total bilirubin (mg/dL)	1.12 (±0.4)	1.15 (±0.4)	1.08 (±0.4)	0.174
Direct bilirubin (mg/dL)	0.35 (±0.4)	0.35 (±0.4)	0.35 (±0.4)	0.956
Indirect bilirubin (mg/dL)	0.77 (±0.4)	0.80 (±0.4)	0.73 (±0.4)	0.203
Prothrombin time (seconds prolonged)	6.3 (±1.2)	6.3 (±1.4)	6.3 (±1.1)	0.669
Ascites	0 (0%)	0 (0%)	0 (0%)	NA
Child–Pugh score, A6/B7/B8/B9	10 (4.7%)/72 (33.6%)/128 (59.8%)/4 (1.9%)	6 (5.3%)/36 (31.9%)/69 (61.1%)/2 (1.8%)	4 (4%)/36 (35.6%)/59 (58.4%)/2 (2%)	0.917
ALBI grade, 1/2/3	20 (9.3%)/186 (86.9%)/8 (3.8%)	10 (8.8%)/99 (87.6%)/4 (3.6%)	10 (9.9%)/87 (86.1%)/4 (4%)	0.950
Cirrhosis	213 (99.5%)	113 (100%)	100 (99%)	0.289
Total cholesterol (mg/dL)	215.9 (±53.9)	215.6 (±53.8)	216.3 (±54)	0.924
Triglycerides (mg/dL)	105.5 (±22)	105.6 (±22)	105.3 (±22)	0.921
Platelet count (No. ×103/μL)	134.2 (±49.5)	121.1 (±39.6)	148.9 (±55.2)	<0.001
Hemoglobin (g/dL)	11.5 (±1.43)	11.6 (±1.42)	11.4 (±1.46)	0.553
White blood cell count (per μL)	4649.4 (±823.5)	4589 (±791.9)	4717 (±856.3)	0.247
Neutrophil count (per μL)	3342.4 (±774.6)	3374.4 (±759.8)	3306.6 (±793.1)	0.531
Lymphocyte count (per μL)	896.9 (±321.6)	784.5 (±300.6)	1022.6 (±298)	<0.001
Monocyte count (per μL)	247.2 (±81.2)	250.3 (±88)	243.8 (±73.2)	0.378
Lymphocyte-to-monocyte Ratio (LMR)	4.20 (±2.27)	3.75 (±2.32)	4.70 (±2.11)	<0.001
Neutrophil-to-lymphocyte Ratio (NLR)	4.43 (±2.61)	5.12 (±2.88)	3.66 (±2.02)	<0.001
Platelet-to-lymphocyte Ratio (PLR)	169.46 (±88.01)	179.15 (±94.86)	158.62 (±78.71)	0.272
Maximum tumor size (cm)	4.49 (±1.13)	4.60 (±1.18)	4.38 (±1.08)	0.371
Bilobar disease	85 (39.7%)	40 (35.4%)	45 (44.6%)	0.172
Capsule	112 (52.3%)	62 (54.9%)	50 (49.5%)	0.433

**Table 3:** Outcomes data grouped by LMR.

Variables	All Patients (*n* = 214)			
		Group 1 Low LMR (*n* = 70)	Group 2 High LMR (*n* = 144)	*p* Value
Technical success	214 (100%)	70 (100%)	144 (100%)	NA
Tumor Response CR PR SD PD	20 (9.3%) 51 (23.8%) 97 (45.3%) 46 (21.5%)	0 (0%) 10 (14.3%) 40 (57.1%) 20 (28.6%)	20 (13.9%) 41 (28.5%) 57 (39.6%) 26 (18.1%)	<0.001
Complete Response	20 (9.3%)	0 (0%)	20 (13.9%)	0.001
Objective Response (CR + PR)	71 (33.2%)	10 (14.3%)	61 (42.4%)	<0.001
Sustained Response Duration ≥ 6 months	111 (51.9%)	38 (54.3%)	73 (50.7%)	0.662
Overall Survival at 6 months	214 (100%)	70 (100%)	144 (100%)	NA
Progression-free survival at 6 months	140 (65.4%)	32 (45.7%)	108 (75%)	<0.001
Successful Downstaging at 6 months	22 (10.3%)	0 (0%)	22 (15.3%)	0.001
Adverse Events	66 (30.8%)	24 (34.3%)	42 (29.2%)	0.447

**Table 4:** Outcomes data, grouped by NLR.

Variables	All Patients (*n* = 214)			
		Group 1 High NLR (*n* = 113)	Group 2 Low NLR (*n* = 101)	*p* Value
Technical success	214 (100%)	113 (100%)	101 (100%)	NA
Tumor Response CR PR SD PD	20 (9.3%) 51 (23.8%) 97 (45.3%) 46 (21.5%)	9 (8%) 16 (14.2%) 58 (51.3%) 30 (26.5%)	11 (10.9%) 35 (34.7%) 39 (38.6%) 16 (15.8%)	0.002
Complete Response	20 (9.3%)	9 (8%)	11 (10.9%)	0.463
Objective Response (CR + PR)	71 (33.2%)	25 (22.1%)	46 (45.5%)	<0.001
Sustained Response Duration ≥ 6 months	111 (51.9%)	55 (48.7%)	56 (55.4%)	0.322
Overall Survival at 6 months	214 (100%)	113 (100%)	101 (100%)	NA
Progression-free survival at 6 months	140 (65.4%)	61 (54%)	79 (78.2%)	<0.001
Successful Downstaging at 6 months	22 (10.3%)	13 (11.5%)	9 (8.9%)	0.533
Adverse Events	66 (30.8%)	38 (33.6%)	28 (27.7%)	0.350

**Table 5:** Logistic regression analysis (Simple–Multiple) of predictive factors affecting Objective Response occurrence.

Predictors	Coeff.	Std. Err.	Wald	*p* > |z|
Age (years)	0.052–0.068	0.013–0.017	17.464–16.714	<0.001–<0.001
Sex (female)	−0.011	0.304	0.001	0.972
Hepatitis C virus	−0.922–−1.858	0.312–0.422	8.714–19.382	0.003–<0.001
α-Fetoprotein (ng/mL)	−0.004–−0.004	0.001–0.001	14.285–11.734	<0.001–0.001
Albumin (g/L)	0.058	0.053	1.204	0.272
White blood cell count (per μL)	<0.001	<0.001	0.193	0.661
Neutrophil count (per μL)	<0.001	<0.001	0.898	0.343
Lymphocyte count (per μL)	0.003–0.004	0.001–0.001	26.029–33.849	<0.001–<0.001
Monocyte count (per μL)	−0.005–−0.004	0.002–0.002	6.091–3.398	0.014–0.065
Neutrophil-to-lymphocyte ratio (NLR)	−0.503	0.108	21.585	<0.001
NLR Groups (<4.72)	1.080	0.302	12.772	<0.001
Lymphocyte-to-monocyte ratio (LMR)	0.322	0.069	21.615	<0.001
LMR Groups (≥2.24)	1.484	0.381	15.172	<0.001
Bilobar disease	−0.562	0.307	3.350	0.067

**Table 6:** Logistic regression analysis (Simple–Multiple) of predictive factors affecting Progression-free Survival at 6 months.

Predictors	Coeff.	Std. Err.	Wald	*p* > |z|
Age (years)	−0.013	0.011	1.477	0.224
Sex (female)	−0.177	0.304	0.339	0.560
Hepatitis C virus	−0.213	0.290	0.541	0.462
α-Fetoprotein (ng/mL)	−0.002–−0.001	0.001–0.001	6.332–2.286	0.012–0.131
Albumin (g/L)	−0.009	0.053	0.031	0.860
White blood cell count (per μL)	<0.001	<0.001	4.335	0.037
Neutrophil count (per μL)	−0.001–<0.001	<0.001–<0.001	8.588–2.286	0.003–0.131
Lymphocyte count (per μL)	0.003–0.003	0.001–0.001	29.031–21.985	<0.001–<0.001
Monocyte count (per μL)	−0.016–−0.014	0.003–0.003	39.167–26.015	<0.001–<0.001
Neutrophil-to-lymphocyte ratio (NLR)	−0.427	0.082	26.832	<0.001
NLR Groups (<4.72)	1.119	0.306	13.353	<0.001
Lymphocyte-to-monocyte ratio (LMR)	0.768	0.121	40.453	<0.001
LMR Groups (≥2.24)	1.270	0.308	17.062	<0.001
Bilobar disease	0.034	0.294	0.013	0.908

## Data Availability

The data presented in this study are available on request from the corresponding author. The data are not publicly available due to privacy issues.

## Abbreviations

AFP: alpha-fetoprotein; ALC: absolute lymphocyte count; AUC: area under the curve; BCLC: Barcelona-Clinic Liver Cancer; CI: Confidence Interval; CLD: chronic liver disease; CR: complete response; cTACE: conventional TACE; CTL: cytotoxic T lymphocyte; DEB: drug-eluting beads; DEM: drug-eluting microspheres; HCC: hepatocellular carcinoma; IL: interleukin; LMR: lymphocyte-to-monocyte ratio; LRTs: locoregional therapies; MLR: monocyte-to-lymphocyte ratio; MRI: magnetic resonance imaging; NLR: neutrophil-to-lymphocyte ratio; OR: objective response; OS: overall survival; PD: progressive disease; PFS: progression-free survival; PLR: platelet-to-lymphocyte ratio; PR: partial response; RFA: radiofrequency ablation; RFS: recurrence-free survival; ROC: receiver operating characteristic; SD: stable disease; SE: standard error; SRD: sustained response duration; TACE: transcatheter arterial chemoembolization; TAMs: tumor-associated macrophages; TGF-β: transforming growth factor-β; TILs: tumor-infiltrating lymphocytes; TME: tumor microenvironment; VEGF: vascular endothelial growth factor.
